# Promoting Effects of the Adipokine, Apelin, on Diabetic Nephropathy

**DOI:** 10.1371/journal.pone.0060457

**Published:** 2013-04-05

**Authors:** Bao-hai Zhang, Wenying Wang, Hongxia Wang, Jiming Yin, Xiang-jun Zeng

**Affiliations:** 1 Department of Pathophysiology, Capital Medical University, Beijing, China; 2 Division of Cardiology, Beijing Tiantan Hospital, Capital Medical University, Beijing, China; 3 Department of Urology, Beijing Friendship Hospital, Capital Medical University, Beijing, China; 4 Beijing Institute Of Hepatology, Beijing You An Hospital, Capital Medical University, Beijing China; University of Tor Vergata, Italy

## Abstract

Angiogenesis, increased glomerular permeability, and albuminuria are thought to contribute to the progression of diabetic nephropathy (DN). Apelin receptor (APLNR) and the endogenous ligand of APLNR, apelin, induce the sprouting of endothelial cells in an autocrine or paracrine manner, which may be one of the mechanisms of DN. The aim of this study was to investigate the role of apelin in the pathogenesis of DN. Therefore, we observed apelin/APLNR expression in kidneys from patients with type 2 diabetes as well as the correlation between albuminuria and serum apelin in patients with type 2 diabetes. We also measured the proliferating, migrating, and chemotactic effects of apelin on glomerular endothelial cells. To measure the permeability of apelin in glomerular endothelial cells, we used transwells to detect FITC-BSA penetration through monolayered glomerular endothelial cells. The results showed that serum apelin was significantly higher in the patients with type 2 diabetes compared to healthy people (*p*<0.05, Fig. 1B) and that urinary albumin was positively correlated with serum apelin (R = 0.78, *p*<0.05). Apelin enhanced the migration, proliferation, and chemotaxis of glomerular endothelial cells in a dose-dependent manner (*p*<0.05). Apelin also promoted the permeability of glomerular endothelial cells (*p*<0.05) and upregulated the expression of VEGFR2 and Tie2 in glomerular endothelial cells (*p*<0.05). These results indicated that upregulated apelin in type 2 diabetes, which may be attributed to increased fat mass, promotes angiogenesis in glomeruli to form abnormal vessels and that enhanced apelin increases permeability via upregulating the expression of VEGFR2 and Tie2 in glomerular endothelial cells.

## Introduction

Nephropathy is a major complication of type 2 diabetes [1]. Glomerular abnormalities are observed in early diabetic nephropathy, including increases in the glomerular filtration rate and albuminuria. These pathological indices are, in part, the consequence of glomerular capillary damage [2]. Previous studies have demonstrated that increased glomerular filtration surface area in diabetic nephropathy is associated with the formation of new glomerular capillaries [3], [4] and a slight elongation of preexisting capillaries [3], [4].

The cellular components of the glomerular capillary are endothelial cells and podocytes, which together with the basement membrane constitute the glomerular filtration barrier. The highly specialized glomerular endothelium contributes to the selective glomerular barrier [5]. Thus, it seems plausible but has not yet been shown that endothelial dysfunction is causally related to the impairment of the glomerular filtration barrier and diabetic nephropathy (DN).

Apelin, a newly discovered adipocytokine [6], is expressed in a variety of tissues, including kidney and endothelial cells [7], [8]. In addition, increased body mass in type 2 diabetic patients might contribute to increased apelin levels in the blood [9]. *In vitro* studies have revealed that apelin and the apelin receptor (APLNR) can induce the sprouting of endothelial cells in an autocrine or paracrine manner, thus suggesting a role for apelin in angiogenesis [10]–[14]. Therefore, apelin may play an important role in DN.

The present study was prompted by a recent report suggesting that the incubation of glomerular endothelial cells with apelin changes myosin light chain (MLC) phosphorylation in vascular smooth muscle cells (VSMCs) under ADMA-induced endothelial leakage conditions [15]. Here, we examined whether DN was associated with the elevation of apelin/APLNR expression in diabetic nephrons and glomerular endothelial cells and whether apelin and APLNR were promoting factors for microalbuminuria in DN.

## Materials and Methods

### Patients

The study protocol was approved by the ethics committee of the Capital Medical University, and informed consent was given by each patient before enrollment. The consent was verbal because the samples were the residues of clinical test specimens. Thus, no extra treatment of the patients was required. The patients signed a sheet that presented the selected cases in table format. The ethics committee of Capital Medical University approved this consent process.

We enrolled 60 patients (31 males with an average age of 55.5±10.6 years and a BMI of 30.8±3.04 kg/m^2^, 29 females with an average age of 54.6±8.6 years and a BMI of 28.7±4.9 kg/m^2^) with type 2 diabetes and 32 clinically healthy subjects (16 males with an average age of 53.1±14.0 years and BMI of 29.6±2.1 kg/m^2^, 16 females with an average age of 54.7±10.3 years and a BMI of 27.4±6.4 kg/m^2^ ) from the outpatient pool. The patients were hospitalized, and their mean duration of diabetes was 12.3±4.1 years. Type 2 diabetes was diagnosed according to the Report of the Expert Committee on the Diagnosis and Classification of Diabetes Mellitus [16]. Exclusion criteria included cardiovascular diseases, cerebrovascular diseases, hypertension, metabolic diseases, and inflammatory diseases.

### Determination of Serum Apelin

The blood samples for the determination of fasting apelin were drawn from all subjects after a minimum of 12 h of overnight fasting. The blood samples were kept in tubes and centrifuged at 3500×*g* to obtain serum samples. The serum samples were separated, aliquoted, stored at −80°C, and assayed within 2 weeks.

Apelin-13 concentrations were measured using a radioimmunoassay kit (Phoenix Pharmaceutical, St. Joseph, MO, USA ). The intra-assay and inter-assay coefficients of variation were 3.8 and 12.5%, respectively. This kit was specific for apelin-13 and had no cross-reactivity to apelin-17 or apelin-36. The lowest detection limit was 20 pg/ml. Apelin-13 was used in this study to verify the function of endogenous apelin-13 on diabetic nephrons.

### Measurement of Albumin in 24-h Urine Samples

Urine samples were taken for biochemical analyses after an overnight fast of 12 h. Urinary albumin was determined in an early morning spot urine sample. The urine albumin concentration was estimated by a double antibody radioimmunoassay (Diagnostic, Los Angeles, CA, US. sensitivity of 0.3 µg/ml) with intra-assay and inter-assay coefficients of variation of 2.7 and 3.5%, respectively.

### Animals and Experimental Design

All procedures were approved by and performed in accordance with the Animal Care and Use Committee of Capital Medical University (20100610 ). All animals received humane care, and the experimental protocol was approved by the Committee of Laboratory Animals according to institutional guidelines.

Diabetic KK-Ay mice have been frequently used as an animal model for non-insulin-dependent diabetes [17], [18]. The symptoms of this animal model are similar to those of diabetic patients. The mice exhibit metabolic abnormalities, such as an absolute or relative lack of insulin, hyperglycemia, glucose intolerance, and higher lipid levels.

Eight-week-old male KK-Ay and C57BL/6 mice were purchased from Capital Medical University (Beijing, China). The mice were housed in air-conditioned, specific pathogen-free animal quarters with lighting from 8∶00 to 21∶00, and the mice were given unrestricted access to a standard laboratory chow and water throughout the study.

KK-Ay mice were randomly allocated to one of three groups as follows: KK mice (KK group, n = 8); KK mice treated with the specific APLNR receptor antagonist, F13 (Gln-Arg-Pro-Arg-Leu-Ser-His-Lys-Gly-Pro-Met-Pro-Ala) (KK+F13A group, n = 8); and KK mice treated with apelin-13 (Gln-Arg-Pro-Arg-Leu-Ser-His-Lys-Gly-Pro-Met-Pro-Phe) (KK+apelin group, n = 8). Apelin-13 (30 µg/kg.day), F13A (25 µg/kg.day), or saline was intraperitoneally injected once a day for 28 days. During the last day of treatment, a urine sample was collected during a 4–10 h period from each mouse housed in a metabolic cage (Tecniplast, Buguggiate, Italy). Albumin and creatine were detected immediately after the sample was collected [19]. The mice were killed under isoflurane anesthesia. Enzyme-linked immunosorbent assay (ELISA) kits were used to measure murine microalbuminuria, and the values were normalized to urinary creatinine (Exocell, Philadelphia, PA, US.).

### Intraperitoneal (i.p.) Glucose Tolerance Test (IGTT) [20]

The intraperitoneal (i.p.) glucose tolerance test (IGTT) was administered to C57BL/6 and KK mice with or without apelin or F13A treatment after the animals were denied access to suckling or food overnight (24 h). Glucose (Wako Pure Chemicals Industries, Osaka, Japan) was administered to the mice by i.p. injection (1 mg/g body weight). Blood samples were collected by tail vein before (0 min) and 30, 60, 90, and 120 min after glucose administration. The blood glucose levels were determined using an automated blood glucose meter (ACCU-CHEK® Performa Nano, Germany).

### Detection of APLNR mRNA with Real-time PCR

To evaluate the APLNR mRNA level, the kidneys were lysed with Trizol reagent, and total RNA was extracted. One microgram of total RNA was reverse-transcribed into single-strand cDNA with M-MuLV reverse transcriptase and random primers. Primers were designed for APLNR (forward, 5′-CCACCTGGTGAAAACTCTCATCA -3′; and reverse, 5′-TGACATAACTGATGCAGG TGC-3′) and glyceraldehyde-3-phosphate dehydrogenase GAPDH (forward, 5′-CTCATGACCAC AGTCCATGC -3′; and reverse, 5′-CACATTGGGGGTAGGAACAC-3′) by Primer Express Software. Real-time PCR was performed in a 25 µL reaction mixture prepared with SYBR GREEN PCR Master Mix (Warrington, UK) containing an appropriately diluted cDNA solution and 0.2 mmol/L of each primer at 95°C for 10 min, followed by 40 cycles at 95°C for 15 s and 60°C for 45 s. Real-time PCR reactions were analyzed by the ABI 7700 Prism Sequence Detection System (PE-ABI). Each tissue sample was run in triplicate. The house keeping gene, GAPDH, was used as an internal control. **ΔΔ**Ct was calculated for each sample.

### Immunohistochemical Assay for the Expression of Apelin-13 in the Kidney

The kidneys were removed from the control (C57BL/6, n = 6) and diabetic (KK mice, n = 6) mice. Tissue sections (5 µm) were deparaffinized and rehydrated by passage through xylene and graded ethanol solutions. The slides were then treated with 1% H_2_O_2_ in PBS for 15 min followed by microwave antigen retrieval at 100°C for 10 min in target retrieval solution (Dako, Carpinteria, CA) in a microwave processor. After sequential 15-min incubations with 0.1% avidin and 0.01% biotin (Vector Laboratories, Burlingame, CA) to block endogenous avidin and biotin, respectively, the slides were then incubated in 0.05% casein with 0.05% Tween 20 in phosphate-buffered saline (PBS) for 30 min to block nonspecific protein binding. The tissues were then incubated with rabbit anti-apelin-13 [pGlu1, Ala13] serum that recognizes all carboxyl-terminal fragments (Phoenix Pharmaceuticals, Belmont, CA) at a 1∶300 dilution for 60 min. Rabbit Ready-to-Use serum (InnoGenex, San Ramon, CA) was used as the negative control. After a PBS wash, the sections were incubated with a biotinylated secondary antibody (goat anti-rabbit IgG; Millipore, Carrigtwohill, Co. Cork, Ireland) at a 1∶300 dilution for 30 min, and signals were detected by streptavidin-horseradish peroxidase colorized by diaminobenzidine (Dako, Carpinteria, CA).

### Cell Culture [21]

Glomeruli were isolated from the renal cortex of adult male C57BL/6 mice (18–22 g). The animals were obtained from the laboratory animal center of Capital Medical University (Beijing, China). All animal procedures were carried out under a protocol approved by the Institutional Animal Care and Use Committee (IACUC) at Capital Medical University. Mice were decapitated under ether anesthesia, and the kidneys were removed and placed into PBS containing NaCl (137 mmol/l), KCl (2.68 mmol/l), KH_2_PO_4_ (1.76 mmol/l), and Na_2_HPO_3_ (0.01 mmol/l) at pH 7.3–7.4. The cortex slices were mashed by differential sieving as previously described [5]. The isolated glomeruli were then incubated at 37°C with 0.1% collagenase type IV (Sigma, St. Louis, MO, USA) for 15 min. The cell suspension was centrifuged at 800×g for 5 min. The precipitate was resuspended in M199 medium (Invitrogen, Grand Island, N.Y. 14072, USA) supplemented with 20% FBS (Hyclone, Logan, Utah, USA), 100 U/ml penicillin, 100 g/ml streptomycin, and 75 ug/ml ECGS (Sigma, St. Louis, MO, USA), and the mixture was then transferred into 1% gelatin-coated (Sigma, St. Louis, MO, USA ) 25-cm^2^ flasks in a humidified incubator at 37°C under 5% CO_2_ and 95% air. The cells were trypsinized with 0.25% trypsin–EDTA (Invitrogen, Grand Island, N.Y. 14072, USA) and plated into flasks, and the medium was replaced every 3 days. The cultured cells were identified by morphological observation and positive staining with antibodies raised against VWF. The third to fifth passages of the glomerular endothelial cells were used in subsequent experiments.

### MTT Assay for Cell Viability

Cell viability was evaluated by the 3-(4,5-dimethyl-2-thiazolyl)-2, 5-diphenyl-2H-tetrazolium bromide (MTT) uptake assay. Because the conversion of MTT into formazan depends on the activity of mitochondrial dehydrogenase, it can be used as an indicator of cell metabolic activity. The MTT assay was performed as previously described [11]. The glomerular endothelial cells were collected and seeded at a density of 8.0×10^3^ cells/well into 96-well flat-bottomed culture plates, and the cells were partially starved in M199 medium supplemented with 1% FBS for 24 h. After reaching sub-confluence, the cells were exposed to various doses of apelin-13 (Sigma, St. Louis, MO, USA) at 37°C for 24 h. In this study, apelin-13 was diluted with the culture medium (M199) to concentrations of 0 M, 10 pM, 1 nM, 100 nM, and 1 µM. Then, 20 µl of MTT working solution (5 mg/ml in PBS) was added to each well of the cultured cells, and the cells were incubated for 4 h at 37°C in humidified air supplemented with 5% CO_2_. The formazan was then solubilized with 150 µl of dimethylsulfoxide (DMSO). The absorbance was detected at an OD of 492 nm with a microplate reader (Wellscan MK3, Labsystems Dragon). Each experiment was performed three times to validate the results.

### BrdU Incorporation Assay for Proliferation

The bromodeoxyuridine (BrdU) incorporation assay was used as a measure of DNA synthesis. Glomerular endothelial cells were partially starved in M199 medium supplemented with 1% FBS for 24 h, and then different concentrations of apelin-13 and BrdU (3000 µg/L) were added. The cells were then incubated for 24 h at 37°C in humidified air supplemented with 5% CO_2_. Glomerular endothelial cells were fixed with 10% formalin in PBS. Fixed glomerular endothelial cells were subsequently treated with 2 N HCl for 30 min and incubated with a mouse monoclonal anti-BrdU antibody (Sigma, St. Louis, MO), and the cells were then labeled with a donkey polyclonal antibody to sheep IgG-H&L (FITC; Abcam, 168 Connaught Road Central, Hong Kong). Cell proliferation was quantified by the average ratio of green nuclear-stained cells to total cells per field in five fields per 3.5-cm dish under a fluorescence microscope at 100× magnification. The experiment was repeated at least three times.

### Cell Motility Assay

The chemotactic motility was assessed using a Boyden chamber with an 8-µm-pore polycarbonate filter (Millipore, 290 Concord Road, Billerica, MA). Fresh assay medium containing different concentrations of apelin-13 was placed in the lower wells. Trypsin-harvested cells (2×10^4^) in 100 µl of the assay medium were loaded into each upper well, and the chamber was then incubated for 24 h. Non-migrating cells on the upper surface of the filter were removed by wiping with a cotton swab. Cell motility was quantified by counting cells that migrated across the filter towards the lower surface in five fields per filter at 200× magnification.

### Migration Scratch-wound Assay

Glomerular endothelial cells were seeded (6×10^4^/well) into 6-well chambers and grown to 100% confluence. The cells were then synchronized in M199 medium supplemented with 1% FBS for 24 h. Confluent cell monolayers were wounded by pressing a sterile 1000-µl pipette tip down onto the plate to cut the cell sheet and to make a sharp visible demarcation at the wound edge (wounds were approximately 1 mm wide) on the plate. The wounded monolayers were washed three times in PBS to remove cell debris and incubated for 24 h at 37°C. M199 medium with 1% FBS (control group) and different concentrations of apelin-13 were added to the wells, and the cells were incubated at 37°C with 5% CO_2_. Three representative images from each well of the scratched areas under each condition were photographed at 0 (immediately after addition of the drug), 5, 22, and 27 h to estimate the migration distance. The experiments were performed at least three times.

### Endothelial Permeability Assay

The endothelial permeability to macromolecules was assessed by measuring the passage of fluorescein isothiocyanate-labeled bovine serum albumin (FITC-BSA; Sigma, St. Louis, USA) across the monolayer. Polycarbonate micropore membranes (diameter of 13 mm and pore size of 0.4 mm; Corning Costar, Cambridge, MA) were gelatinized (type II calf skin gelatin). Endothelial cells (1×10^5^ in 0.50 ml of M199 medium) were then seeded onto the gelatinized membranes, and the cells were cultured for 5–7 days (37°C and 5% CO_2_) to allow the cells to become confluent. In brief, the system consists of two compartments separated by a microporous polycarbonate membrane lined with the endothelial cell monolayer. The luminal (upper) compartment (0.7 ml) was suspended in the abluminal (lower) compartment (2.5 ml). Cells in both wells and the insert containing glomerular endothelial cell monolayers were partially starved in M199 medium supplemented with 1% FBS. After one h, the medium in the insert was replaced with 500 µl of M199 medium with 1% FBS containing 0.5 mg/ml FITC-BSA. The medium in the well was then replaced with 600 µl of M199 medium with 1% FBS containing various concentrations of apelin-13 (0 M, 10 pM, 1 nM, 100 nM, and 1 µM). At one, two, and three h, 200-µl aliquots were removed, and the same amount of M199 medium with 1% FBS containing various concentrations of apelin-13 was added. The fluorescence of the aliquots was measured using a fluorospectrometer at 492/520 nm absorption/emission wavelengths for FITC-BSA. The concentration of FITC-BSA was calculated in reference to a set of standard dilutions.

### Western Blotting

Cells were seeded in 6-well plates, grown to 90% confluence, and starved in M199 medium supplemented with 1% FBS for 24 h, which was followed by stimulation with different concentrations of apelin (0 M, 10 pM, 1 nM, 100 nM, and 1 µM) for 24 h. The cells were washed with ice-cold PBS, lysed with lysis buffer (50 mM Tris–HCl, pH 6.8; 1% Tween 20; 0.25% sodium deoxycholate; 150 mM NaCl; 1 mM EDTA; 1 mM Na3VO4; 1 mM NaF; 4% SDS; 0.2% bromophenol blue; 20% glycerol; and complete protease inhibitor cocktail) (Roche Diagnostics GmbH, Mannheim, Germany), and boiled for 5 min. The protein concentration was measured by a bicinchoninic acid assay (Bio-Rad, Munich, Germany). Aliquots of samples (50 µg/lane) were resolved by 10% sodium dodecyl sulfate–polyacrylamide gel electrophoresis (SDS–PAGE) and transferred to nitrocellulose membranes. After blocking with 5% nonfat milk, the membranes were probed with primary antibodies (VEGFR2, 1∶2000; and Tie2, 1∶1000; Cell Signaling Technologies, Beverly, MA, USA) at 4°C overnight, followed by incubation with the corresponding horseradish peroxidase-conjugated secondary antibody (1∶2000; Amersham Pharmacia Biotech, Freiburg, Germany). Equal loading was controlled with an anti-mouse GAPDH monoclonal antibody (1∶5000; Sigma–Aldrich GmbH, Taufkirchen, Germany). Visualization was performed using the enhanced chemiluminescence detection system (ECL; Amersham Pharmacia Biotech) according to the manufacturer’s specifications. Chemiluminescence was captured on Kodak X-ray film. Signals were quantified using a BioDoc Analyzer (Goettingen, Germany).

### Statistical Analysis

The results were expressed as mean values ± SD. Significant differences between groups were analyzed using unpaired two-tailed Student’s t-tests. All analyses were performed using the SPSS 11.0 statistical software package for Windows (SPSS Inc., Chicago, USA). A *p-*value less than 0.05 was considered statistically significant.

## Results

### Correlation between Apelin and Albuminuria in DN

**Figure 1:**
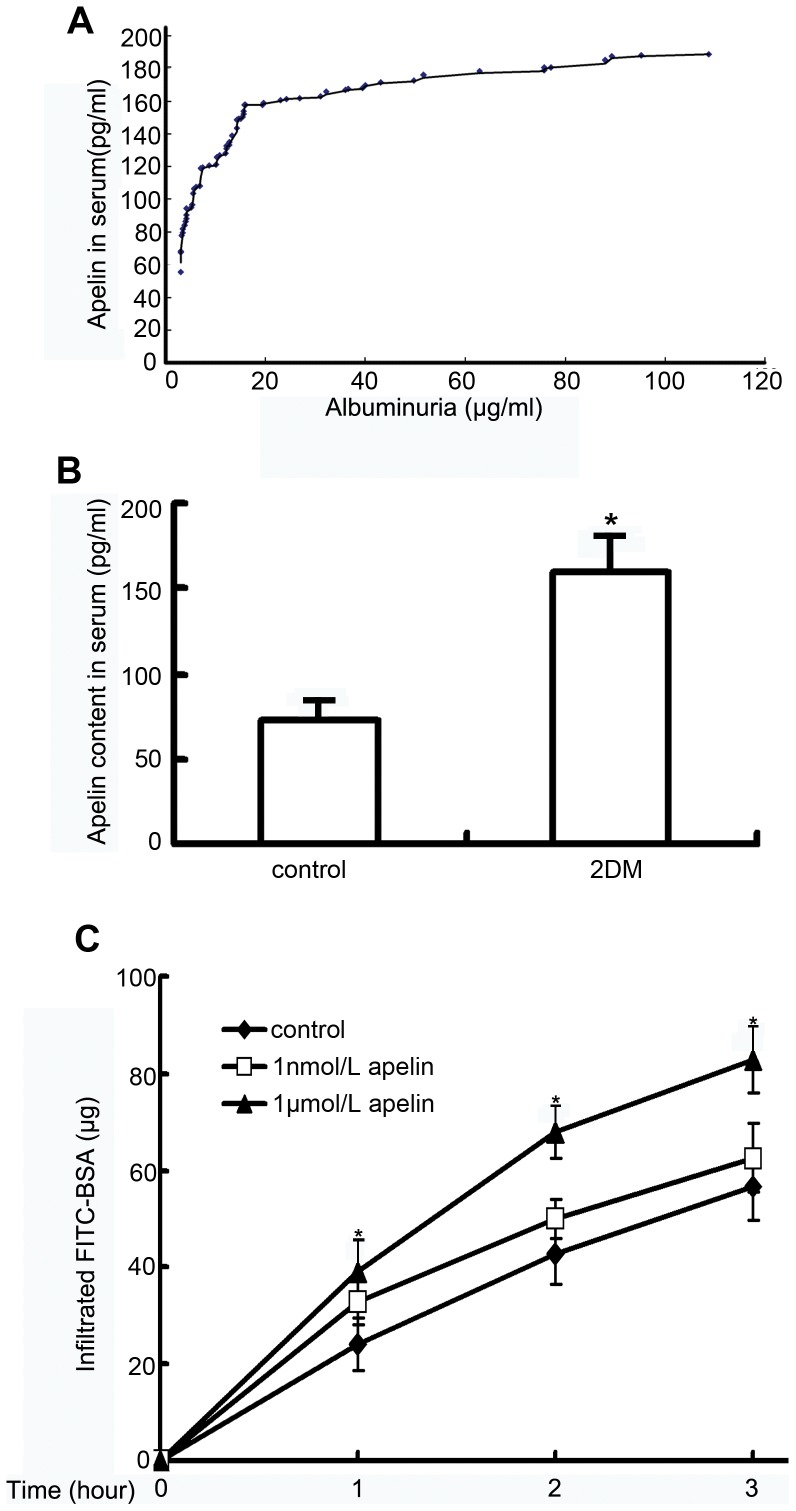
Correlation between apelin and albuminuria. A: The apelin concentration in serum was positively correlated with albuminuria (R = 0.78, *p<0.05). B: The apelin concentration in serum was significantly increased in patients with type 2 diabetes (2DM, n = 60) compared to healthy people (control, n = 32). The data are expressed as the means±SD (*p<0.05 vs. control grou ). C: The graphs show the promoting effect of apelin on FITC-BSA passing through the glomerular endothelial cell monolayers at the indicated time point. The data are expressed as the means±SD (n = 6, *p<0.01 vs. control group).

Albuminuria is usually an early symptom of DN. To examine the role of apelin in albuminuria, we determined the correlation between serum apelin and albuminuria. The results revealed that albuminuria increased as serum apelin increased (R = 0.78, *p*<0.05, Fig. 1A ). In addition, higher levels of apelin in patients with type 2 diabetes were observed. The apelin concentrations were 160±21 pg/ml (n = 60) in the type 2 diabetic group and 72±12 pg/ml (n = 32) in the control group (*p*<0.05, Fig. 1B).

**Figure 2:**
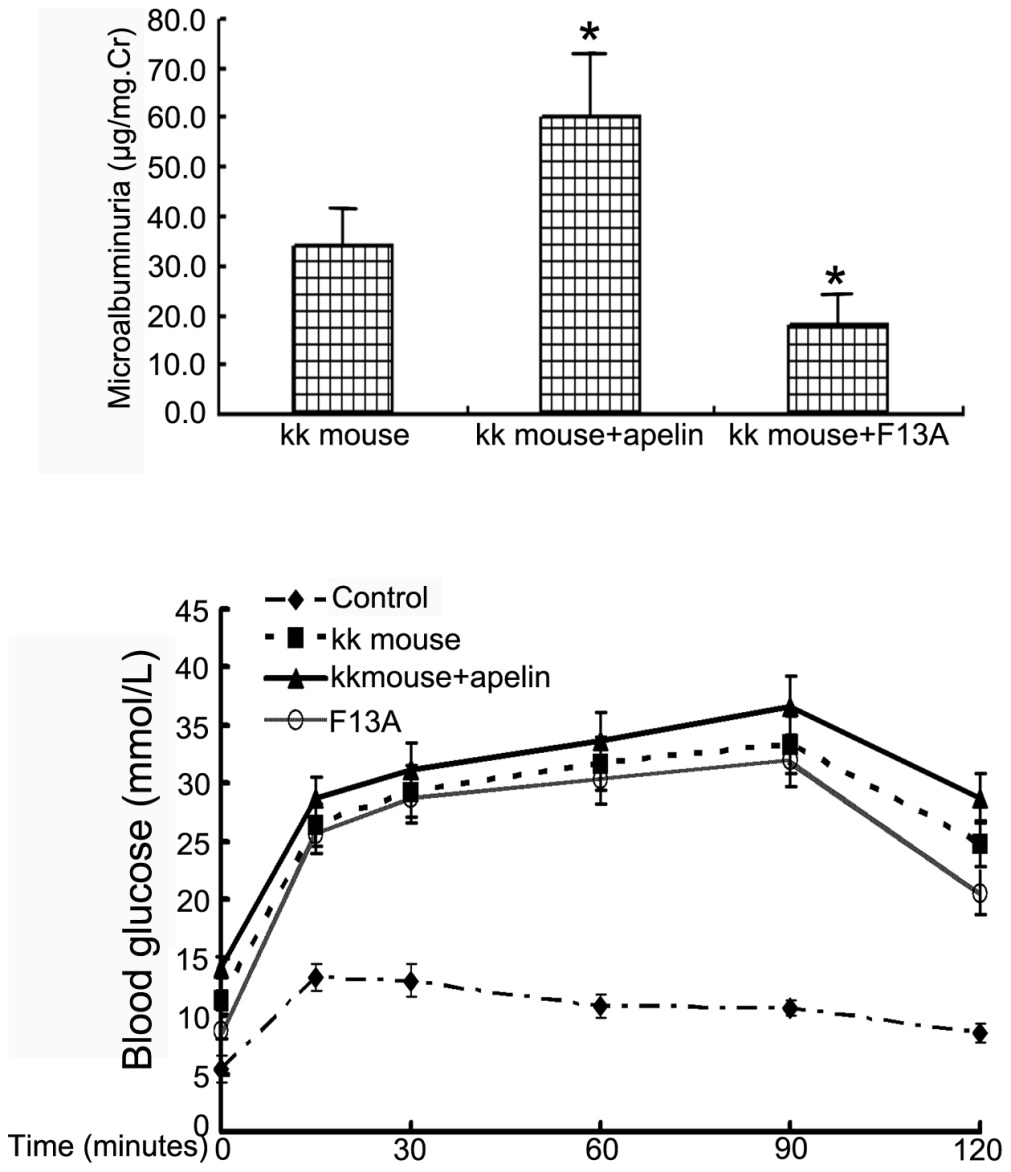
Promoting effects of apelin on microalbuminuria and glucose concentration in blood. A: Apelin and the antagonist of apelin, F13A, were injected into KK mice for 4 weeks. At the end of the experiments, urine was collected and microalbumin and creatinine were detected. Microalbumin/mg Cr increased from 34.3±6.9 µg/mg Cr in KK mice to 59.2±13.6 µg/mg Cr in apelin-treated KK mice and decreased to 18.6±4.7 µg/mg Cr in F13A-treated KK mice (n = 8, *p<0.05 vs. KK mice). B: For the intraperitoneal (i.p.) glucose tolerance test (IGTT), blood glucose levels were measured at the indicated time points following i.p. The results are expressed as mmol/L glucose in blood. The results are expressed as the means±SEM (n = 8,*p<0.05 vs. C57BL/6 mice).

Urine microalbumin was measured after injecting KK mice (type 2 diabetes model) with apelin-13 or F13A to verify that apelin promoted albuminuria in DN. The results showed that urine microalbumin increased from 34.3±6.9 µg/mg Cr in KK mice to 59.2±13.6 µg/mg Cr after treatment with apelin-13 (n = 8, *p*<0.05. Fig. 2A), and treatment with F13A was associated with a decrease to 18.6±4.7 µg/mg Cr (n = 8, *p*<0.05 vs. KK mice, Fig. 2A). Interestingly, apelin increased the blood glucose concentration, as assessed by the IGTT, and F13A decreased the blood glucose concentration, although the difference was not significant (*p*>0.05, n = 8, Fig. 2B). The glucose concentration in the KK mice was significantly increased compared to that in the C57/BL mice (*p*<0.05, n = 8, Fig. 2B).

### APLNR Expression is Significantly Upregulated in Mice with type 2 Diabetes

**Figure 3:**
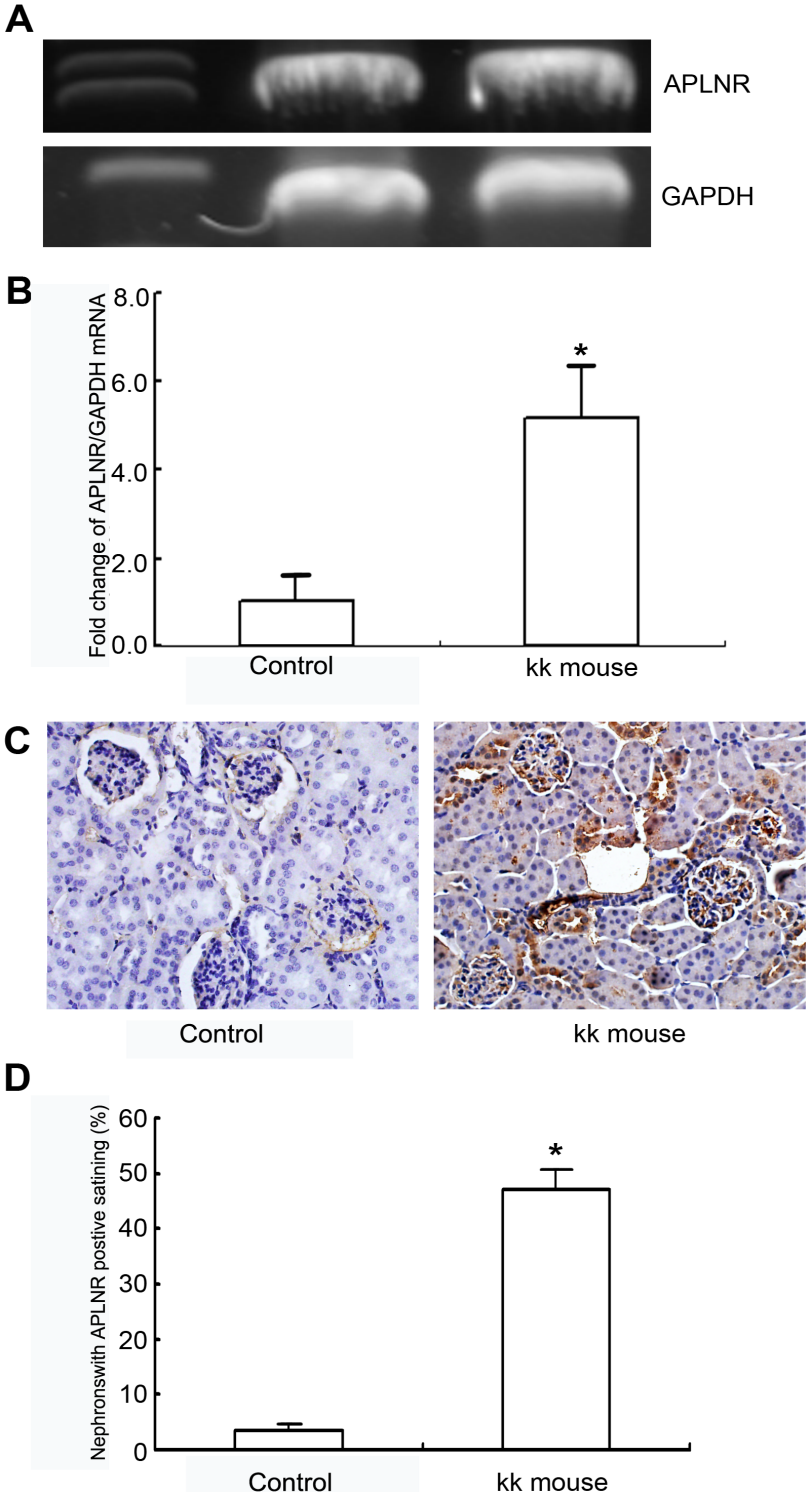
Apelin/APLNR expression in the mouse kidney. A and B: APLNR mRNA was analyzed with real time-PCR. The relative amount of APLNR mRNA in the kidney of type 2 diabetic mice (KK mice) was significantly increased by 5.2-fold compared with that of control mice (C57BL/6). The data are expressed as the means±SD (n = 3, *p<0.01 vs. control group). C and D: The immunohistochemical assay of apelin expression in the kidney showed that the amount of glomeruli with apelin positive staining was significantly higher in type 2 diabetic mice (KK mice) compared to that in control mice (C57BL/6 mice). The bar in C indicates 10 µm. The data are expressed as the means±SD (n = 8, *p<0.01 vs. control group).

Using agarose gel analysis, the molecular weights of the real time-PCR products for the APLNR and GAPDH primer pairs were verified (Fig. 3A). The relative amount of APLNR mRNA in the kidneys of type 2 diabetic mice (KK mice) was increased 5.2-fold compared to the relative mRNA amount in the kidneys of control mice (C57BL/6) (Fig. 3B, n = 3, *p*<0.01 vs. control group).

### Apelin Expression in the Mouse Kidney by Immunohistochemistry

Immunohistochemical analysis detected apelin expression in the glomeruli of mouse kidneys. The percentage of positively stained areas in the glomeruli of mice with type 2 diabetes (48.1±3.4% in the KK mice, n = 8) was significantly higher than in the control mice (3.3±1.1% in the C57BL/6 mice, n = 8) (Figs. 3C and 3D, *p*<0.01).

### Proliferating Effects of Apelin-13 on Glomerular Endothelial Cells

**Figure 4:**
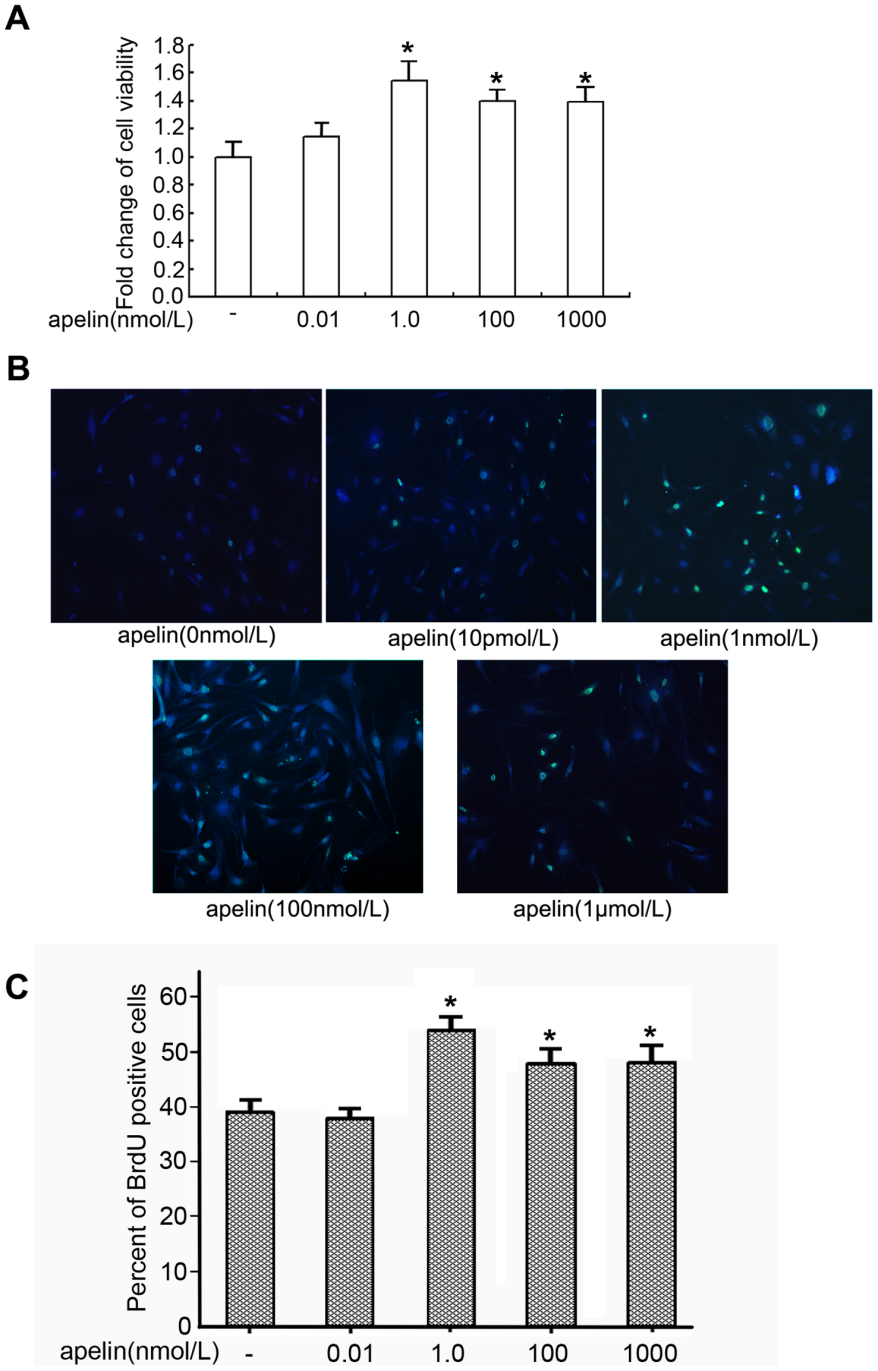
Proliferating effect of apelin on glomerular endothelial cells. A: Glomerular endothelial cells were incubated with apelin-13 (0, 0.01, 1, 100, and 1000 nmol/L ) for 24 h. The MTT assay showed that cell viability was increased significantly at the concentration of 1 nM apelin-13 compared to the control group. The data represent the mean±SD (n = 6, *p<0.05 vs. control group; 0 nmol/L apelin). B and C: The BrdU incorporation assay demonstrated that apelin promoted the incorporation of BrdU into glomerular endothelial cells in a dose-dependent manner after incubation for 24 h. The data are expressed as the means±SD (n = 6, *p<0.05 vs. control group).

We examined the viability of glomerular endothelial cells using the MTT assay. After exposure for 24 h, apelin increased the cell viability of glomerular endothelial cells in a dose-dependent manner, and 1 nM apelin caused a significant increase in cell viability (1.54-times more than the control group; *p*<0.05 vs. control group, n = 6, Fig. 4A). The BrdU incorporation assay was used to examine the proliferation of glomerular endothelial cells. After a 24 h exposure to apelin, 51.4±2.4% of the glomerular endothelial cells treated with 1.0 nmol/L apelin stained positive for BrdU, and only 39.4±2.1% of the untreated glomerular endothelial cells stained positive for BrdU (Figs. 4B and 4C, n = 6, *p*<0.05). These results suggested that pathologically elevated levels of apelin in diabetes promote the proliferation of glomerular endothelial cells. However, apelin in higher concentrations may be toxic to the cells.

### Effects of Apelin on Glomerular Endothelial Cell Migration

**Figure 5:**
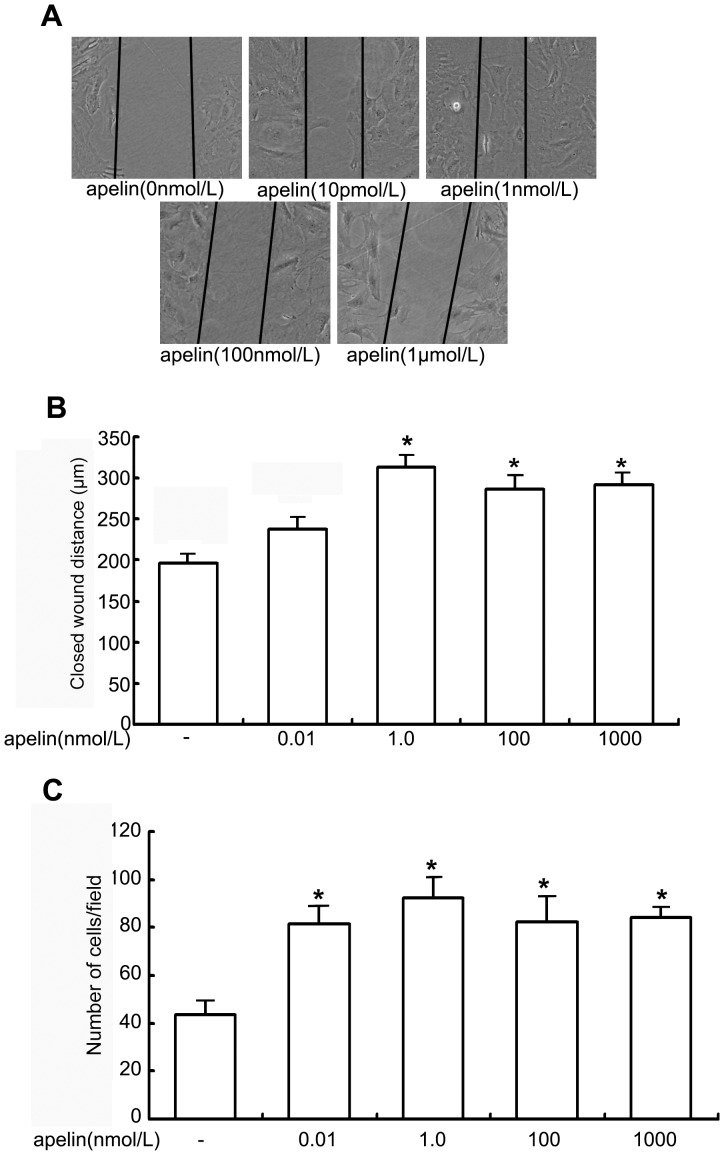
Migrating and chemotactic effects of apelin on glomerular endothelial cells. A: The scratch-wound assay for glomerular endothelial cells was performed in the presence of apelin. The injury was performed by scraping the monolayer (the denuded area is between the panel lines). After 24 h of incubation in the presence or absence of apelin, cell migration into the wound edge was observed by light microscopy. The distance migrated to the denuded area is shown in graph. B: The decreased distance to the denuded area was significantly enhanced by apelin-13 at the concentration of 1 nmol/L compared to that in the control group (apelin at a concentration of 0 nmol/L). The data are expressed as the means±SD (n = 6, *p<0.05 vs. control group). C. Chemotactic effect of apelin on glomerular endothelial cells. The data are expressed as the means±SD (n = 6, *p<0.05 vs. control group).

We examined the effects of apelin on the migration of glomerular endothelial cells using a scratch assay. After exposure to apelin for 24 h, glomerular endothelial cells treated with 1 nmol/L apelin-13 migrated 314±14 µm into the wound, and untreated cells migrated 192±12 µm into the wound (n = 6, *p*<0.05, Figs. 5A and 5B). These results suggested that apelin in higher concentrations stimulates the glomerular endothelial cells to migrate, which may contribute to the progression of DN.

### Chemotactic Effects of Apelin on Glomerular Endothelial Cells

We measured the chemotactic effects of apelin on glomerular endothelial cells using a transwell assay. Apelin (concentrations ranging from 0.01 nmol/L to 1.0 µmol/L) promoted the movement of glomerular endothelial cells through the transwell from 82±8 cells to 92±9 cells, but only 43±6 untreated cells moved through the transwell without apelin treatment(n = 6, *p*<0.05, Fig. 5C). These results suggested that the elevated apelin levels in DN stimulate the glomerular endothelial cells to migrate.

### Apelin Increases Permeability of Glomerular Endothelial Cells

Apelin has been reported to regulate vascular permeability. Therefore, we examined how apelin changes the permeability of glomerular endothelial cells. We analyzed endothelial permeability in the presence of various concentrations of apelin using FITC-BSA. FITC-BSA passing through the glomerular endothelial cell monolayer increased with time. After one h, only 23±3 µg of FITC-BSA passed through the transwell without apelin treatment, but 32±3 µg of FITC-BSA (1 nmol/L) and 38±5 µg of FITC-BSA (1 µmol/L) passed through the transwell with apelin treatment. After two h, 41±4 µg of FITC-BSA passed through the transwell without apelin treatment, but 49±3 µg of FITC-BSA (1 nmol/L) and 68±4 µg of FITC-BSA (1 µmol/L) passed through the transwell with apelin treatment. After three h, 57±6 µg of FITC-BSA passed through the transwell without apelin, but 62±5 µg of FITC-BSA (1 nmol/L) and 82±6 µg of FITC-BSA (1 µmol/L) passed through the transwell with apelin treatment (n = 6, *p*<0.01, Fig. 1C). These results indicated that the permeability of glomerular endothelial cells is enhanced by apelin in DN. Therefore, elevated apelin may contribute to albuminuria in DN.

### Effects of Apelin on VEGFR2/Tie2 Expression in Glomerular Endothelial Cells

**Figure 6:**
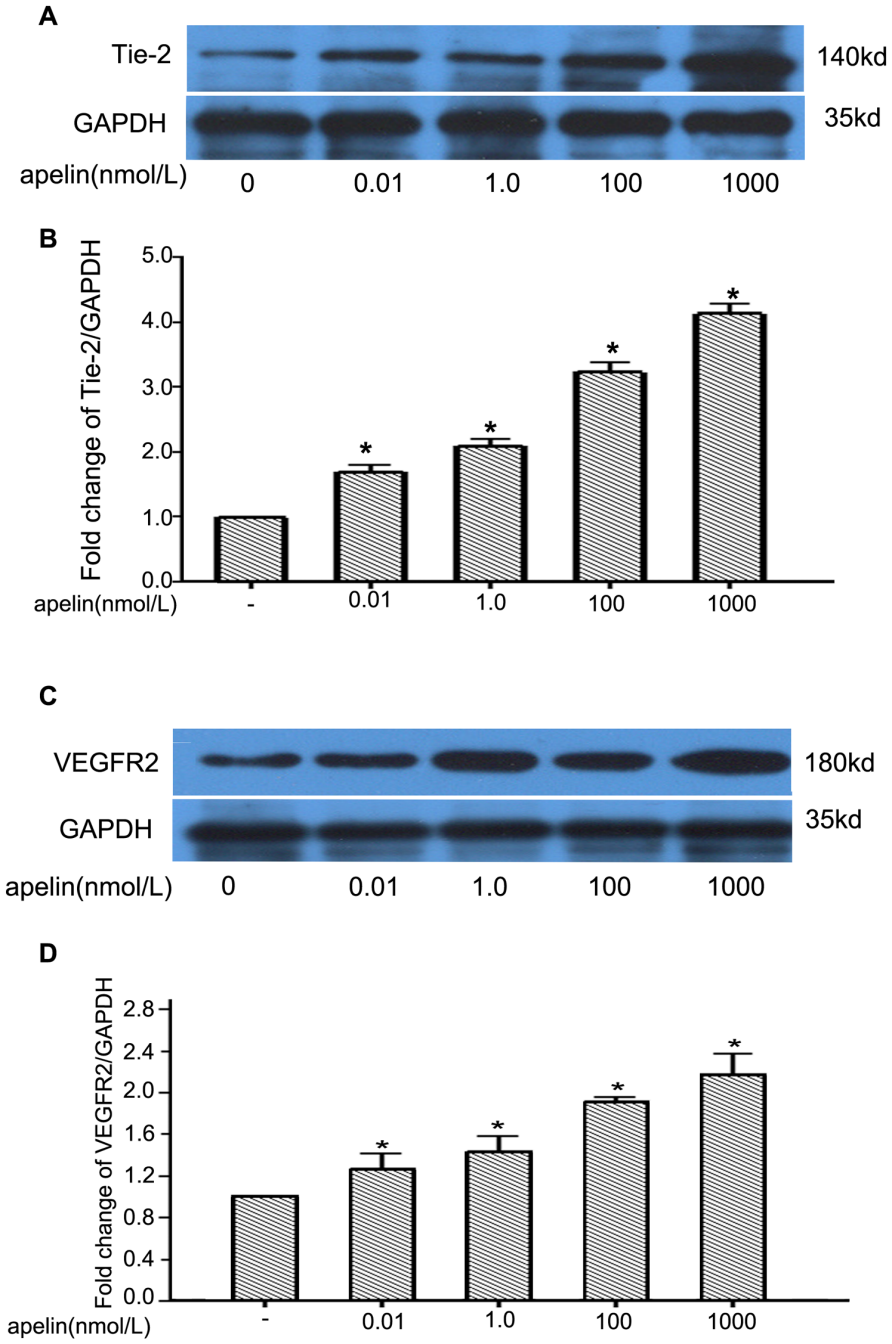
Western blotting analyses of Tie2 and VEGFR2 in cultured glomerular endothelial cells. A: The images are derived from identical gels of Tie2 and GAPDH, and each lane was loaded with the same amount of protein (50 µg ). The numbers indicate the expected molecular weights of the bands. B: Densitometry of Tie2 levels normalized to GAPDH for each treatment condition in A. The data are expressed as the means±SD (n = 3, *p<0.05 vs. control group ). C: Images are derived from identical gels of VEGFR2 and GAPDH, and each lane was loaded with the same amount of the protein (50 µg ). D: Densitometry of VEGFR2 levels normalized to GAPDH for each treatment condition in C. The data are expressed as the means±SD (n = 3, *p<0.05 vs. control group).

To examine the effect of apelin on the expression of VEGFR2 and Tie2, confluent glomerular endothelial cells were exposed to apelin-13 (0–1 µM) for 24 h. As shown in Figs. 6A and 6B, apelin increased the protein expression levels of Tie2 by 1.72±0.17-, 2.12±0.13-, 3.34±0.21-, and 4.13±0.18-fold at concentrations of 0.01, 1.0, 100 and 1000 nmol/L, respectively, compared to control cells (n = 3, *p*<0.01 vs. control group). Similarly, the effect of apelin on the expression of VEGFR2 was investigated by treating glomerular endothelial cells with 0–1 µmol/L apelin. Following 24 h of exposure to exogenous apelin, VEGFR2 levels were significantly increased by 1.25±0.21-, 1.45±0.18-, 1.82±0.06-, and 2.07±0.32-fold at concentrations of 0.01, 1.0, 100 and 1000 nmol/L, respectively, compared to the levels in the control cells (n = 3, *p*<0.01 vs. control group) (Figs. 6C and 6D). These results suggested that elevated apelin in DN promotes angiogenesis in glomeruli by the upregulation of VEGFR2 and Tie2.

## Discussion

The common early signs of diabetic nephropathy (DN) are microalbuminuria and overt proteinuria. Our results indicated that serum apelin levels are positively correlated with microalbuminuria in patients with type 2 diabetes (Fig. 1A). We were interested in determining if apelin in serum was higher in patients with type 2 diabetes, and we found that patients with type 2 diabetes had higher apelin concentrations compared to healthy people (Fig. 1B). These results indicated that apelin/APLNR might be a promoting factor for DN.

Therefore, we studied the effects of apelin-13 and the antagonist of apelin-13, F13-A, on the microalbumin concentrations in urine and glucose concentrations in blood. The results indicated that the ratio of microalbumin to creatinine in urine was increased by apelin and was decreased by F13A (Fig. 2A). These results suggested that apelin/APLNR plays a promoting role in the development of DN. Interestingly, apelin showed a tendency to increase blood glucose in KK mice, and F13A had the opposite effect on blood glucose concentration (Fig. 2B). These results suggested that apelin may regulate glucose metabolism in diabetes.

In addition, we detected apelin and APLNR expression in the kidneys of type 2 diabetic mice (KK mice). Apelin/APLNR expression was significantly increased in KK mice (Figs. 3A and 3B). Based on these results, we hypothesized that an obesity-induced apelin increase in type 2 diabetes might contribute to glomerular capillary injury and diabetic nephropathy.

It is generally accepted that endothelial dysfunction is important for diabetic microvascular disease [22]. However, there has been little direct evidence of a causative link among endothelial dysfunction, microvascular disease, and diabetic end-organ damage. Microvascular disease can lead to organ damage through impaired vascular function, increased inflammation, or increased apoptosis [23]–[25]. The growth of new blood vessels and increased permeability of microvessels in nephrons are believed to be the main pathogenesis of DN [26]. In the current study, glomerular endothelial cells incubated with apelin were more permeable to FITC-BSA (Fig. 1C). The observed increase of glomerular permeability with apelin may, therefore, be relevant during the early stages of DN, thereby supporting the concept that endothelial dysfunction is causally linked to DN.

Previous *in vitro* studies have shown that apelin contributes to tube formation in RF/16A cells [12]. Histological changes to blood vessels in glomeruli are found in patients with type 1 or 2 diabetic nephropathy [27]–[29]. Swollen endothelial cells and thin walls in the basement membrane of abnormal vessels in diabetics have been observed, suggesting that they are structurally immature [29]–[31]. Abnormal vessels are associated with increased glomerular hypertrophy and enhanced frequency of glomerular occlusion, fibrinoid lesions, tubulointerstitial injury, and urinary albumin excretion [28], [32], [33]. Blocking angiogenesis attenuates glomerular basement membrane thickening and mesangial expansion [34]–[36], thereby indicating that the increase in abnormal vessels contributes to the development of early features of DN.

If apelin induces glomerular capillary sprouting to form structurally immature vessels, then the proliferation of glomerular endothelial cells will be the first step. Therefore, we measured the proliferating effects of apelin on glomerular endothelial cells. MTT and BrdU assays revealed that apelin induced the proliferation of glomerular endothelial cells in a dose-dependent manner (Figs. 4A–4C). These findings clearly suggested that apelin may be a crucial factor for pathological glomerular angiogenesis.

Apelin performs angiogenic functions by endocrine or paracrine pathways. Glomerular endothelial cells have to migrate to the sites of apelin secretion to form new capillaries. We investigated the chemotactic and migratory effects of apelin on glomerular endothelial cells. The results showed that apelin accelerated wound healing in glomerular endothelial cells (Fig. 5A) and increased the number of migrated cells as measured by chemotaxis (Fig. 5B). These results confirmed that apelin, as an endocrine or paracrine peptide, facilitates abnormal vessel formation in diabetic glomeruli, which helps DN progress. Glomerular hyperfusion and hyperfiltration usually occur in the early stages of DN. Affluent arterioles appear to be more dilated than efferent arterioles. These early hemodynamic changes alleviate albumin leakage from glomerular capillaries, overproduction of the mesangial cell matrix, thickening of the glomerular basement membrane, and injury to podocytes. Several factors, such as angiotensin II, nitric oxide (NO), prostanoids, vascular endothelial growth factor (VEGF), and TGF- ß1, have been reported to affect the irregular autoregulation in DN [37]. Apelin causes nitric oxide-dependent arterial dilation *in vivo* in humans [38]. Both VEGFR2 and Tie2 are principally expressed in endothelial cells. In the present study, we detected an upregulation of VEGFR2 and Tie2 by apelin in glomerular endothelial cells (Fig. 6). VEGFR2 can promote proliferation and chemotaxis, and it can induce the permeability of endothelial cells by binding to VEGF [39]. In addition, Tie2 can inhibit vascular permeability and tighten preexisting vessels [40], and it plays a critical role in the angiogenesis of endothelial cells via binding to angiopoietin (Ang) [41]. These results suggested that apelin contributes to the glomerular hyperfusion and hyperfiltration that often occur in the early stages of DN.

In conclusion, the results of the present study identified a previously unknown role for apelin in regulating DN by modulating the permeability and proliferation of glomerular endothelial cells. Apelin-mediated angiogenesis and increased permeability in diabetic glomeruli identified a crucial role of apelin in the pathogenesis of diabetic nephropathy.
